# Establishment of Mouse Model of *MYH9* Disorders: Heterozygous R702C Mutation Provokes Macrothrombocytopenia with Leukocyte Inclusion Bodies, Renal Glomerulosclerosis and Hearing Disability

**DOI:** 10.1371/journal.pone.0071187

**Published:** 2013-08-20

**Authors:** Nobuaki Suzuki, Shinji Kunishima, Makoto Ikejiri, Shoichi Maruyama, Michihiko Sone, Akira Takagi, Masahito Ikawa, Masaru Okabe, Tetsuhito Kojima, Hidehiko Saito, Tomoki Naoe, Tadashi Matsushita

**Affiliations:** 1 Department of Hematology and Oncology, Nagoya University Graduate School of Medicine, Showa-ku, Nagoya City, Aichi Prefecture, Japan; 2 Department of Advanced Diagnosis, Clinical Research Center, National Hospital Organization Nagoya Medical Center, Naka-ku, Nagoya City, Aichi Prefecture, Japan; 3 Department of Medical Technology, Nagoya University School of Health Sciences, Higashi-ku, Nagoya City, Aichi Prefecture, Japan; 4 Department of Nephrology, Nagoya University Graduate School of Medicine, Showa-ku, Nagoya City, Aichi Prefecture, Japan; 5 Department of Otorhinolaryngology, Nagoya University Graduate School of Medicine, Showa-ku, Nagoya City, Aichi Prefecture, Japan; 6 Department of Experimental Genome Research, Genome Information Research Center, Osaka University, Suita City, Osaka Prefecture, Japan; 7 Honorary director, National Hospital Organization Nagoya Medical Center, Naka-ku, Nagoya City, Aichi Prefecture, Japan; 8 Department of Transfusion Medicine, Nagoya University Hospital, Showa-ku, Nagoya City, Aichi Prefecture, Japan; Institut National de la Santé et de la Recherche Médicale, France

## Abstract

Nonmuscle myosin heavy chain IIA (NMMHCIIA) encoded by *MYH9* is associated with autosomal dominantly inherited diseases called *MYH9* disorders. *MYH9* disorders are characterized by macrothrombocytopenia and very characteristic inclusion bodies in granulocytes. *MYH9* disorders frequently cause nephritis, sensorineural hearing disability and cataracts. One of the most common and deleterious mutations causing these disorders is the R702C missense mutation.

We generated knock-in mice expressing the *Myh9* R702C mutation. R702C knock-in hetero mice (R702C+/− mice) showed macrothrombocytopenia. We studied megakaryopoiesis of cultured fetal liver cells of R702C+/− mice and found that proplatelet formation was impaired: the number of proplatelet tips was decreased, proplatelet size was increased, and proplatelet shafts were short and enlarged. Although granulocyte inclusion bodies were not visible by May–Grünwald Giemsa staining, immunofluorescence analysis indicated that NMMHCIIA proteins aggregated and accumulated in the granulocyte cytoplasm.

In other organs, R702C+/− mice displayed albuminuria which increased with age. Renal pathology examination revealed glomerulosclerosis. Sensory hearing loss was indicated by lowered auditory brainstem response.

These findings indicate that *Myh9* R702C knock-in mice mirror features of human *MYH9* disorders arising from the R702C mutation.

## Introduction

May-Hegglin Anomaly (MHA) is an autosomal-dominant inherited disorder characterized by macrothrombocytopenia and Döhle body-like cytoplasmic inclusion bodies in granulocytes. Ten years ago, we and others showed that *MYH9*, which encodes non-muscle myosin heavy chain IIA (NMMHCIIA), is mutated in this disorder [1]–[3]. *MYH9* is expressed in hematological cells, as well as in kidney, cochlea and lens cells. Thus, patients with a *MYH9* mutation often suffer from nephritis, deafness and cataracts. A new disease entity, *MYH9* disorders, has been proposed to encompass a wide variety of clinical phenotypes [4], [5]. To date, more than 40 *MYH9* mutations have been reported. Among these, the R702C mutation is associated with the development of nephritis and hearing loss in young patients [6]–[8].

*Myh9* homozygous knockout mice die at the embryonic stage, while heterozygous knockout mice are phenotypically normal [9], [10]. Thus, simple haploinsufficiency is not the pathogenetic mechanism underlying *MYH9* disorders. Zhang et al. reported three mutant *Myh9* mouse lines, D1424N and E1841K in the tail domain and R702C in the head domain, that reproduced clinical phenotypes in mice. However, R702C hetero mice were generated by disrupting *Myh9* exon 2 and replacing it with human NMMHCIIA harboring R702C fused to eGFP (GFP-R702C mice) [11]. In human *MYH9* disorders, it is known that R702C mutation shows more severe macrothrombocytopenia than other mutations [8], while such mutations as R702C in the head domain, are known to induce severe nephritis [6]. However, the clinical phenotypes of GFP-R702C mice were weaker than anticipated. Here, we have employed a different knock-in strategy with GFP-R702C mice to generate and characterize mice expressing the R702C mutation in the mouse gene. The DNA construct was intended to replace the endogenous *Myh9* gene with an R702C mutation. A Neo marker inserted into the intron upstream of *Myh9* exon 15 is flanked, by loxP sequence, then removed by crossing with a CAG-Cre mouse. Thus we successfully left the *Myh9* DNA sequence as intact as possible, such that R702C+/− mice have only one amino acid substitution of R702C.

The established R702C knock-in hetero mice (R702C+/− mice) are expected to fully express the hematological/non-hematological phenotypes found in patients with *MYH9* disorders as compared with GFP-R702C mice.

## Materials and Methods

### Construction of the *Myh9* R702C knock-in vector

**Figure 1:**
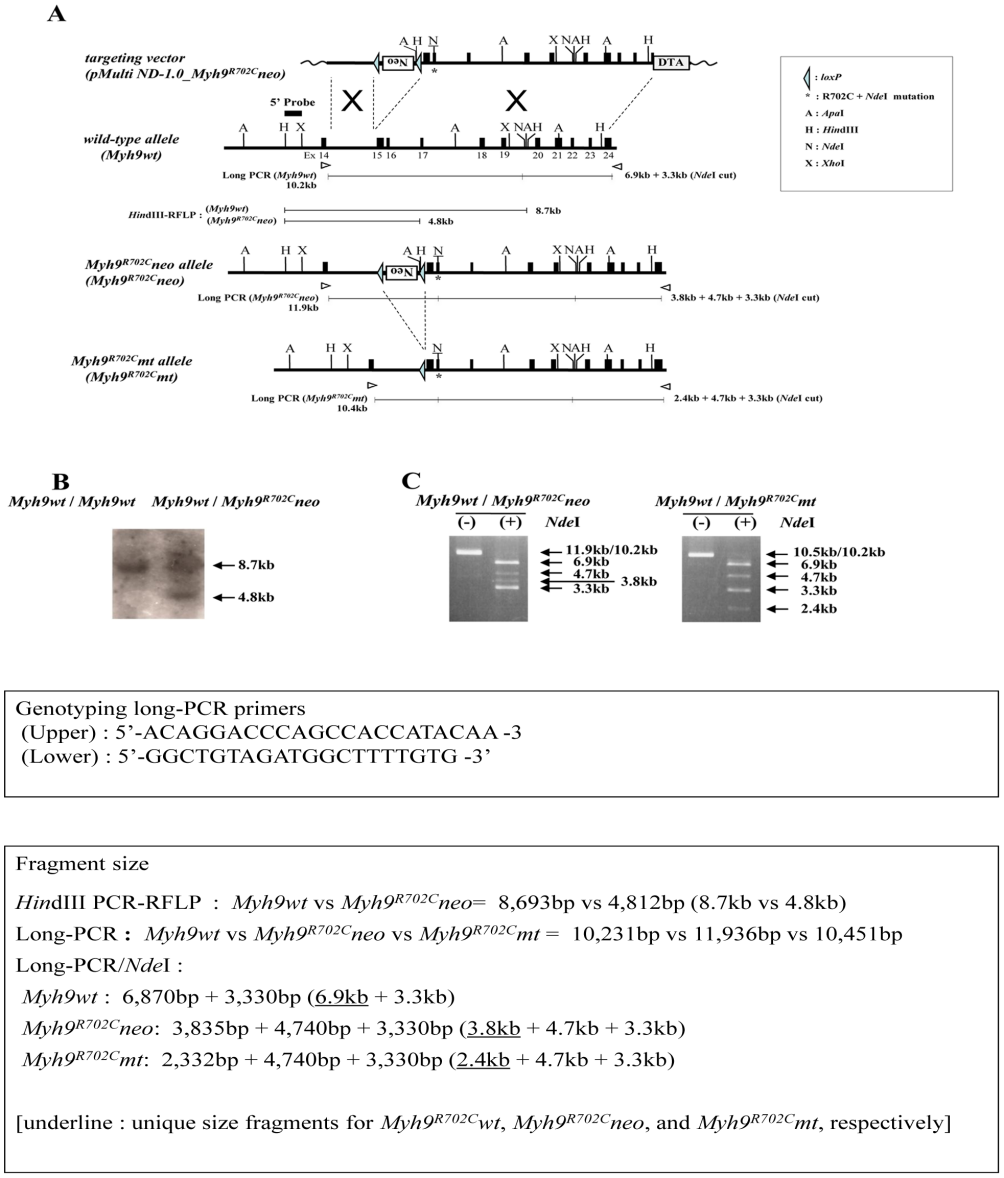
Myh9 R702C knock-in strategy. A) Targeting strategy for R702C knock-in mutation of the murine *Myh9* gene. The targeting vector p*Multi-ND1.0_Myh9^neo^*, the wild-type *Myh9* allele and the targeted allele before (*Myh9^neo^*) and after Cre-mediated excitation of the *loxP* franked *neo* cassette (*Myh9^mt^*) are schematically represented. The targeting vector also contains the Diphtheria toxin fragment A *(DTA)* gene outside the flanking homologies. Black boxes in the genomic structures represent exon sequences; the asterisk on exon 16 denotes the R702C mutation; the underlined N (*Nde*I) denotes the extra diagnostic restriction site created by silent mutation. The expected restriction fragments of the genotyping PCR products are indicated with their relative size accompanied with solid lines. The black box above the wild-type gene represents the 5′ probe used for Southern blot analysis. The open arrowheads under the gene represent the primers used for long-PCR genotyping. (B, C) Confirmation at the DNA level of correct targeting of the *Myh9* gene. Correct homologous recombination as identified by an additional 4.8-kb band in Southern blot analysis of *Hin*dIII digested genomic DNA with the 5′ probe, as well as by a 3.8 kb targeted fragment in long-PCR products digested with *Nde*I (B). Correct Cre-mediated excitation of the *loxP* franked *neo* cassette as confirmed by the appearance of a 2.4 kb recombined instead of 3.8 kb targeted fragment in long-PCR products digested with *Nde*I (C).

A genomic DNA fragment containing murine *Myh9* (C57BL/6J, Accession number NC_000081) was obtained by PCR and used as a probe to isolate a genomic clone containing a segment of *Myh9* from a 129SVJ lambda FIX II genomic library (Stratagene, La Jolla, CA, USA). The targeting vector (pMulti ND-1.0_*Myh9* ^R702C^neo) was constructed from basic vector pMulti ND-1.0 [12]. The *MYh9* fragments consisted of a *Bst*XI/*Bgl*II fragment as the 5′ arm and a *Bgl*II/*Sau*3AI fragment as the 3′ arm (Figure 1A). We introduced the R702C mutation into the 3′ arm and an additional silent mutation to create a diagnostic *Nde*I site.

### Generation of Targeted Mice

Linearized targeting vector was electroporated into D3 ES cells derived from 129Sv and screened for neomycin resistance. Two homologous recombinant ES clones were independently injected into C57B6 blastocysts to generate chimeric mice. Male chimera derived from one ES clone transmitted the recombinant allele to the next generation (*Myh9^R702C^neo*). The loxP-*neo* cassette was removed by crossing the heterozygous mice with a CAG-Cre deleter mouse strain that constitutively expresses Cre recombinase to yield heterozygous knock-in mice (R702C+/− mice). Long-range PCRs were performed using the 5′ external sense primer (5′-ACAGGACCCAGCCACCATACAA) and the 3′ external antisense primer (5′-GGCTGTAGATGGCTTTTGTG), followed by *Nde*I digestion to confirm the mutant (Figure 1C).

All research procedures involving animals were performed in accordance with the Laboratory Animals Welfare Act, the *Guide for the Care and Use of Laboratory Animals*, and the Guidelines and Policies for Rodent Experiments provided by the Institutional Animal Care and Use Committee (IACUC) at the Nagoya University Graduate School of Medicine and were reviewed and approved by the IACUC. The protocol was approved by the committee on the Ethics of Animal experiments of the Nagoya University Graduate School of Medicine (Permit Number:25016).

### Hematological analysis

Whole blood was obtained from the aorta. Complete blood cell counts were determined using an automated blood cell analyzer (SE9000; Sysmex, Kobe, Japan). Blood smears were stained with May–Grünwald Giemsa (MGG) solution for immunofluorescence analysis. Platelet counts of R702C+/− mice were determined manually from MGG-stained smears. Bone marrow cells from dissected femurs were subjected to hematoxylin-eosin (HE) staining and anti-CD41 and NMMHCIIA immunostaining.

### NMMHCIIA immunostaining

Immunofluorescence analysis of granulocyte NMMHCIIA was performed as described previously [13]. Methanol-fixed and acetone-permeabilized blood smears were incubated with anti-NMMHCIIA antibody (BT561; Biomedical Technologies Inc., Stoughton, MA, USA) and reacted with Alexa Fluor 555-labeled goat anti-rabbit IgG (Invitrogen, San Diego, CA, USA). Stained cells were examined under a fluorescence microscope (BX50; Olympus, Tokyo, Japan).

### Tail bleeding time measurement

Mice were anesthetized, the tail was cut 5 mm from the tip, and the tail was immediately immersed into a tube filled with 1 mL saline at 37°C. Bleeding time was defined as the time required for bleeding to stop.

### Clot retraction

Blood was obtained from the aorta of anesthetized mice, and was immediately transferred to a tube containing EDTA. Platelet-rich plasma (PRP) was collected from the supernatant after 40 min of stationary incubation in the tube, and then platelet-poor plasma (PPP) was obtained by centrifugation at 70× g for 5 minutes with no brake. Platelet number of PRP was adjusted to 30×10^4^/µl by dilution with PPP. Mouse erythrocytes (2 µL) were obtained from the centrifuged sediment and added to the PRP suspension (200 µL). Finally, thrombin (final concentration, 10 U/mL; Sigma-Aldrich, Tokyo, Japan) was added in the presence of 20 mM CaCl_2_ and incubated for up to 2 hours at 37°C.

### Culture of fetal liver-derived megakaryocytes

Fetal liver cells were harvested from embryonic day 13.5 embryos and cultured in Dulbecco's modified Eagle medium supplemented with 10% fetal calf serum (FCS) and 50 ng/mL human thrombopoietin, as described previously [14]. Proplatelet formation was monitored in suspension by inverted fluorescence microscopy (IX71; Olympus, Japan), or by cytospin preparations stained with MGG and immunostained with anti-CD41 or NMMHCIIA.

### Evaluation of albuminuria and hematuria

Spot urine samples free of fecal contamination were collected and the concentration of albumin was quantitatively determined using an ELISA kit (AlbuwellM; Exocell, Philadelphia, PA, USA).

Urine samples were separated by sodium dodecyl sulfate-polyacrylamide gel electrophoresis on 4%–12% gradient acrylamide slab gels (Invitrogen, San Diego, CA, USA) under reducing conditions, and gels were then stained with Coomassie Brilliant Blue R-250.

Urine hematuria was analyzed at the age of 20 weeks by using conventional strip method based on the peroxidase activity of hemoglobin (Eiken Chemical, Tochigi, Japan).

### Histological evaluation of kidney

Tissue for light microscopy was fixed in 4% paraformaldehyde phosphate buffer solution, processed, then embedded in paraffin. Three-micrometer sections were stained with periodic acid-methenamine-silver (PAM).

In order to determine the extent of glomerulosclerosis, 10 fields were randomly selected from the renal cortex. The number of glomeruli was counted under a microscope at ×100 magnification (Olympus, Tokyo, Japan), and the number of glomeruli per field was converted number to per mm^2^. The percentage of glomeruli with sclerosis was determined based on the ratio of glomeruli with sclerosis/total glomeruli.

For electron microscopy, small pieces of kidney tissue were fixed in 2.5% glutaraldehyde for 2 hours at 4°C, osmicated, and then embedded in Epon 812 (Nisshin EM, Tokyo, Japan). Ultrathin sections were observed under a JEM-2010 electron microscope (JEOL, Tokyo, Japan).

### Measurement of auditory brain stem response (ABR)

Mice were anesthetized, and then needle electrodes were inserted into the vertex (positive), auricle of the right ear (negative) and auricle of the left ear (ground). Tone bursts of 1-ms duration with a rise and fall time of 0.1 ms at frequencies of 8, 12, 16 and 20 kHz were produced using a sound stimulator (RP2.1; Tucker-Davis Technology Co., Alachua, FL, USA) and a speaker (ES-1; Tucker-Davis Technology Co.) connected to the right ear canal. A total of 512 tone-burst-evoked responses were obtained with amplifier filters set below 100 Hz and above 3 kHz. The mean of the amplified responses was determined using the program PowerLab 4/20 (ADInstruments, Castle Hill, Australia) and displayed on a monitor. Auditory brain stem responses were obtained by decreasing the stimuli in 5-dB steps from a maximum intensity of 100 dB sound pressure level.

## Results

### Generation of R702C+/− mice

In order to investigate the molecular and pathological mechanisms underlying human *MYH9* disorders, we introduced a *Myh9* R702C mutation into the mouse genome using a knock-in approach (Figure 1). Germline transmission of the targeted allele was obtained and identified by Southern blot and long-range PCR analysis. The targeted embryonic stem cells were injected into blastocysts and implanted into surrogate females to generate *Myh9* R702C knock-in chimeric mice. These mice were crossed with B6.Cg-Tg(CAG-cre/Esr1*)5Amc/J (The Jackson Laboratory, Bar Harbor, ME, USA) to excise the floxed Neo resistance cassette.

R702C+/− mice had an extremely low birthrate: only 12.0% by crossing R702C+/− mice and C57BL/6j mice (Table S1). Heterozygous mating yielded no homozygous mutant offspring, suggesting that this is an embryonic lethal phenotype, as in the absence of *Myh9* [10]. We verified that R702C homozygous mice were alive at E12.5, but no living embryos were detected at E18.5 (Table S1).

### R702C+/−mice display macrothrombocytopenia

**Figure 2:**
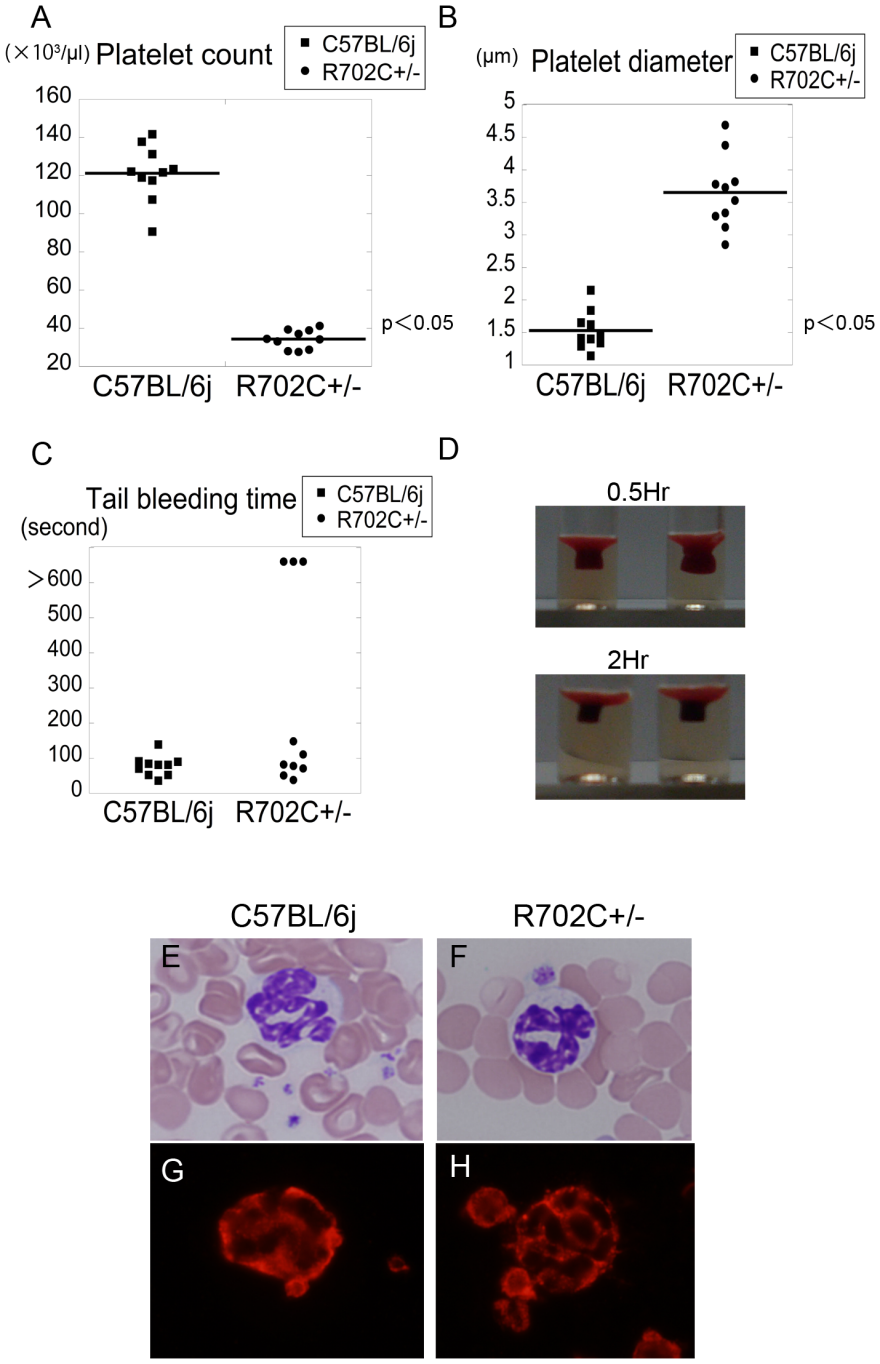
Macrothrombocytopenia and abnormal NMMHCIIA accumulation in granulocytes. R702C+/− mice exhibited decreased platelet count (mean±SD, C57BL/6j: 1212±146×10^9^/L vs. R702C+/−: 342±49×10^9^/L) (n = 10) (A). Platelet size was evaluated by measuring platelet diameter (mean±SD: C57BL/6j: 1.52±0.29 µm vs. R702C+/−: 3.65±0.56 µm) (n = 10) (B). Most R702C+/− mice had a bleed time comparable to WT mice, although several displayed prolonged bleed times (n = 10) (C). Clot retraction was not impaired in R702C+/− mice. Data are representative of three experiments (D). There were no abnormalities in granulocytes and no granulocyte inclusion bodies were visible following May–Grünwald Giemsa (MGG) staining (E) (F). Immunofluorescence analysis showed that NMMHCIIA aggregated and accumulated in the granulocyte cytoplasm of R702C+/− mice (G)(H).

All R702C+/− mice exhibited lower platelet counts than C57BL/6j mice (wild-type mice unrelated to R702C +/− mice: WT mice). An average platelet count represented around 30% of C57BL/6j mice (Figure 2A). The diameter of platelets from R702C+/− mice was more than twice that from WT mice (Figure 2B).

### R702C+/− mice are equivalent to WT mice in bleeding time and clot retraction

R702C+/− mice have no spontaneous bleeding tendency. Three out of 10 R702C+/− mice displayed prolonged bleeding time, but most bled for approximately the same length of time as WT mice (Figure 2C). Clot retraction was slightly reduced in R702C+/− mice, but there were no significant differences when compared with WT mice (Figure 2D).

### Abnormal localization of NMMHCIIA in granulocytes

Although the presence of granulocyte inclusion bodies in conventionally stained blood smears is the most characteristic feature of human *MYH9* disorders, R702 mutations are associated with faint or invisible inclusion bodies [8]. Inclusion bodies were also invisible in R702C+/− mice (Figure 2E and F). However, immunofluorescence analysis for NMMHCIIA revealed an abnormal localization of the protein that we define as type II small punctuated or granular cytoplasmic granules [8] (Figure 2G and H). This indicates that mutant NMMHCIIA displays the same aggregation-prone features in humans and in mice [13].

### Abnormal proplatelet formation is present in cultured fetal liver-derived megakaryocytes from R702C+/− mice

**Figure 3:**
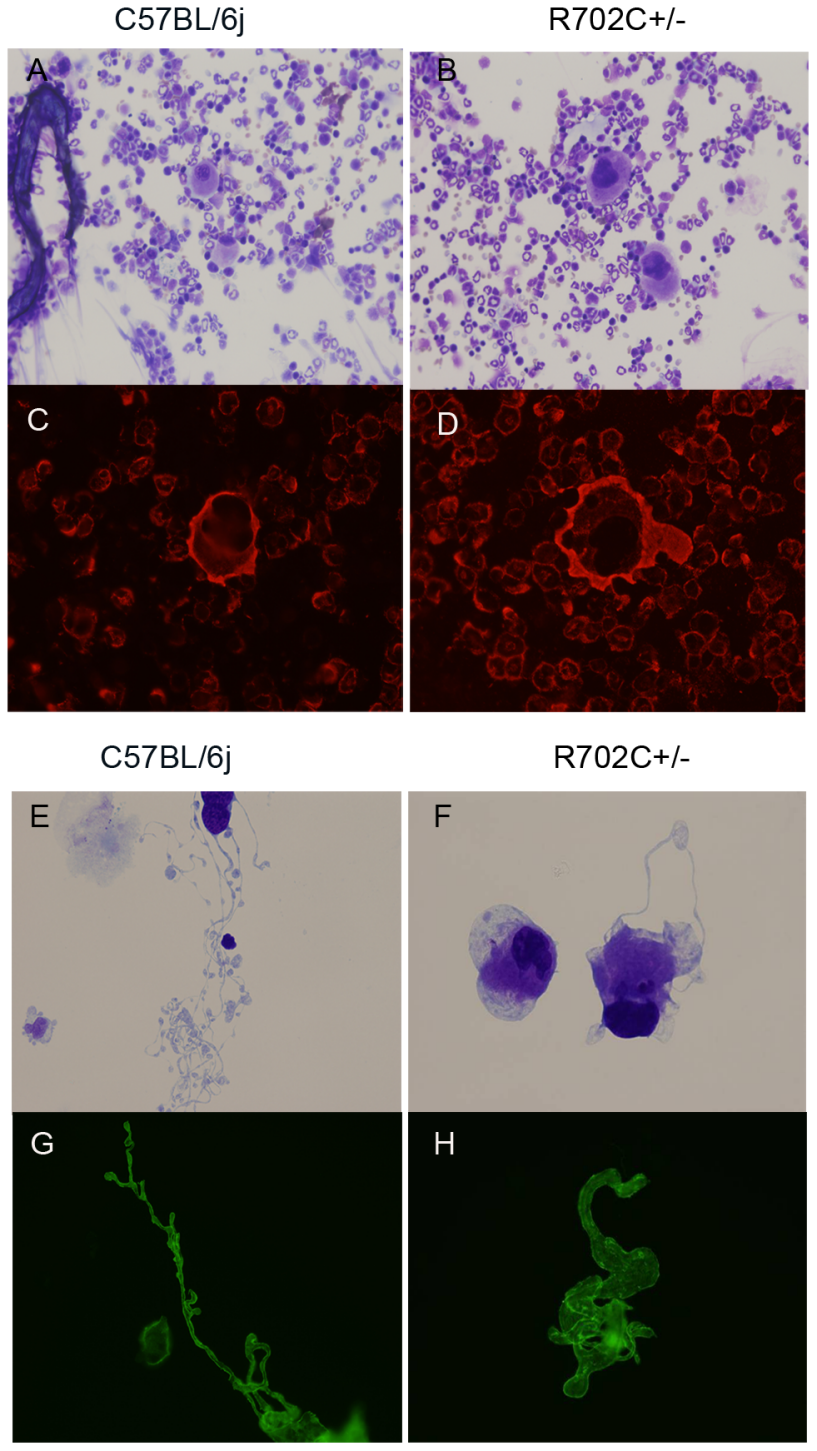
Abnormal megakaryocytopoiesis. In hematoxylin-eosin staining, the morphology of megakaryocyte in R702C+/− mice was not different from that of WT mice (A) (B). There were no significant differences in the distribution of NMMHCIIA between R702C+/− mice and WT mice by immunostaining (C) (D). Proplatelet formation was explored by cultured fetal liver cells. In WT mice, the proplatelet shaft extends from the cell spindle, leading to the formation of proplatelet beads. R702C+/− mice had shorter and thicker shafts when compared with WT mice, and the proplatelet beads were fewer and larger than those of WT mice. MKs were stained with MGG (E) (F) or were observed using CD41 immunofluorescence (G) (H).

We examined the morphology of bone marrow megakaryocytes by MGG staining. Although megakaryocyte number was increased (Figure S1), their morphology was comparable to that of WT mice (Figure 3A and B) and showed no abnormal pattern of NMMHCIIA localization (Figure 3C and D).

We evaluated proplatelet formation using cultured megakaryocytes. The percentage of megakaryocytes with extended proplatelets was significantly lower in R702C+/− mice (Figure S2). In WT mice, the proplatelet shaft that extends from the cell spindle causes the formation of numerous proplatelet beads. R702C+/− mice had shorter and thicker shafts and fewer and larger proplatelet beads when compared to WT mice (Figure 3E–H). These results are consistent with thrombocytopenia and increased platelet size in patients with *MYH9* disorders, indicating that the heterozygous R702C mutation leads to abnormal proplatelet formation.

### R702C+/− mice display progressive albuminuria and glomerulosclerosis

**Figure 4:**
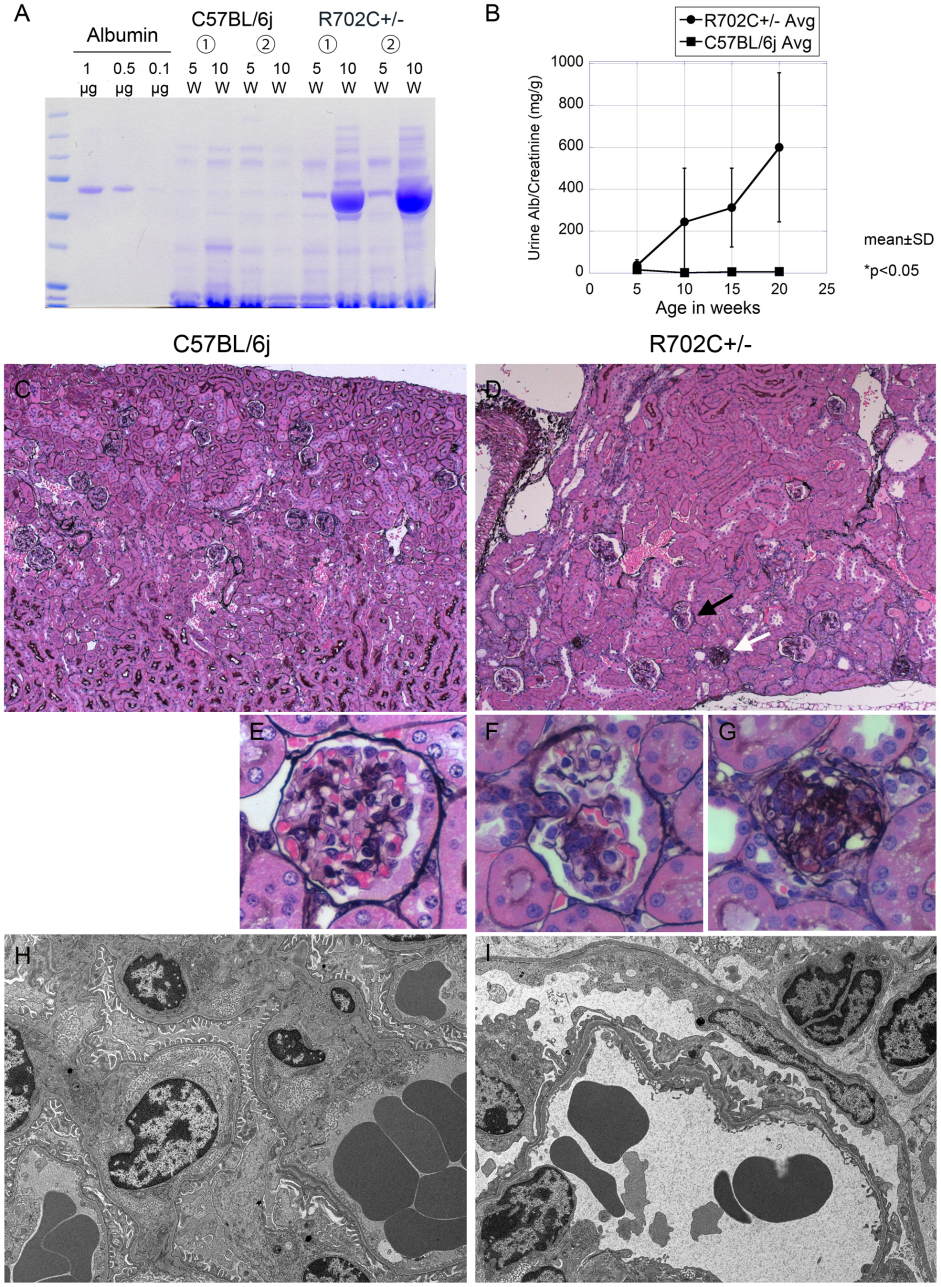
Abnormal kidney function. Coomassie blue-stained SDS-PAGE gel of urine samples from 5 and 20 week old mice (A). Albuminuria was also measured at 5, 10, 15 and 20 weeks using an ELISA kit (AlbuwellM, Exocell) (n = 6) (B). These results show that, while WT mice had little albuminuria at any age, R702C+/− mice had significant albuminuria from the age of 5 weeks. This progressive albuminuria was observed in all R702C+/− mice. Pathologic confirmation of albuminuria was obtained by light and electron microscopy of renal specimens from 20-week-old R702C+/− mice and WT mice. Light microscopy samples were stained with Periodic acid-methenamin (PAM) and were observed at 100-fold magnification (C) (D) and 630-fold magnification (E) (F) (G). R702C+/− mice displayed significant glomerulosclerosis (D), primarily global glomerulosclerosis (indicated by white arrow in D and magnified on (G)) and some segmental glomerulosclerosis (indicated by black arrow in D and magnified on (F)). (C) and (E) are normal controls. Electron microscopic analysis was performed at 3000-fold magnification (H) (I). Transmission electron microscopy revealed foot process effacement (I). (H) is normal control.

R702C+/− mice showed progressive albuminuria with age, while WT mice had trace amounts of urine albumin between 5–20 weeks (Figure 4A and B). At five weeks of age, all R702C+/− mice developed severe albuminuria. However, R702C+/− mice displayed little hematuria from 7 to 20 weeks of age (Table 1).

**Table 1:** Urine examination by urinary test strip.

	R702C +/− (7 weeks)						R702C +/− (20 weeks)						C57BL/6j (20 weeks)					
	1	2	3	4	5	6	1	2	3	4	5	6	1	2	3	4	5	6
Hematuria	−	−	−	−	−	−	−	−	−	−	−	−	−	−	−	−	−	−
Proteinuria	±	++	+	+	++	+	+++	+	+++	++	+++	++	±	−	−	−	±	±
Urinal sugar	−	−	−	−	−	−	−	−	−	−	−	−	−	−	−	−	−	−
PH	6	5	5	5	5	5	5	5	5	5	5	6	6	6	6	6	5	7

Hematuria: − means RBC<10/µl, Hb<0.03 mg/dl.

Proteinuria: ±:15, +:30, ++:100, +++:300 (mg/dl).

Urinal sugar: ±:50, +:100, ++:250, +++:500 (mg/dl).

Kidney pathology from R702C+/− mice was grossly abnormal. Pathological examination by light microscopy revealed glomerular sclerosis. Global glomerulosclerosis was mainly seen (Figure 4C and E), and some glomeruli displayed segmental glomerulosclerosis (Figure 4C and F). Electron microscopy showed podocyte foot process effacement (Figure 4G and H) characteristic of podocyte injury. These findings are compatible with human patients with R702C mutations [7]. Quantification of the percentage of glomeruli with sclerosis was also performed (Table 2). Glomerular changes ranging from mild segmental sclerosis to global sclerosis were observed in all regions of the renal cortex, and accounted for 43.4% of total glomeruli (Table 2).

**Table 2:** Percentage of glomeruli with sclerosis.

	C57BL/6j (20 weeks)		R702C+/− mice (20 weeks)				
	1	2	1	2	3	4	5
Glomerulus with Sclerosis (/field)	0,0,0,0,00,0,0,0,0	0,0,0,0,00,0,0,0,0	7,11,10,7,98,16,6,5,9	6,10,7,9,66,12,7,10,11	4,3,4,9,67,7,8,4,8	6,7,2,5,22,2,3,4,3	6,6,10,13,79,8,3,4,3
Average Glomerulus with Sclerosis (/mm^2^)	0.0	0.0	24.8	23.6	16.9	10.1	19.9
Total Glomerulus (/field)	17,10,1411,10,1012,10,10,16	10,15,1315,15,910,11,10,13	17,21,2614,15,1730,10,915	14,16,916,15,1632,15,1722	14,11,1111,15,1315,18,1618	22,21,1314,12,1310,14,1212	14,11,1422,18,1813 ,9,910
Average Total Glomerulus (/mm^2^)	33.8	34.1	49.0	48.4	40.0	40.3	38.8
Ratio of Glomerulus with Sclerosis (%)	0.0	0.0	50.6	48.8	42.3	25.2	50.0

Mean ratio of glomerulus with sclerosis (%): 43.4% (n = 5).

### Some R702C+/− mice exhibit sensory deafness

**Figure 5:**
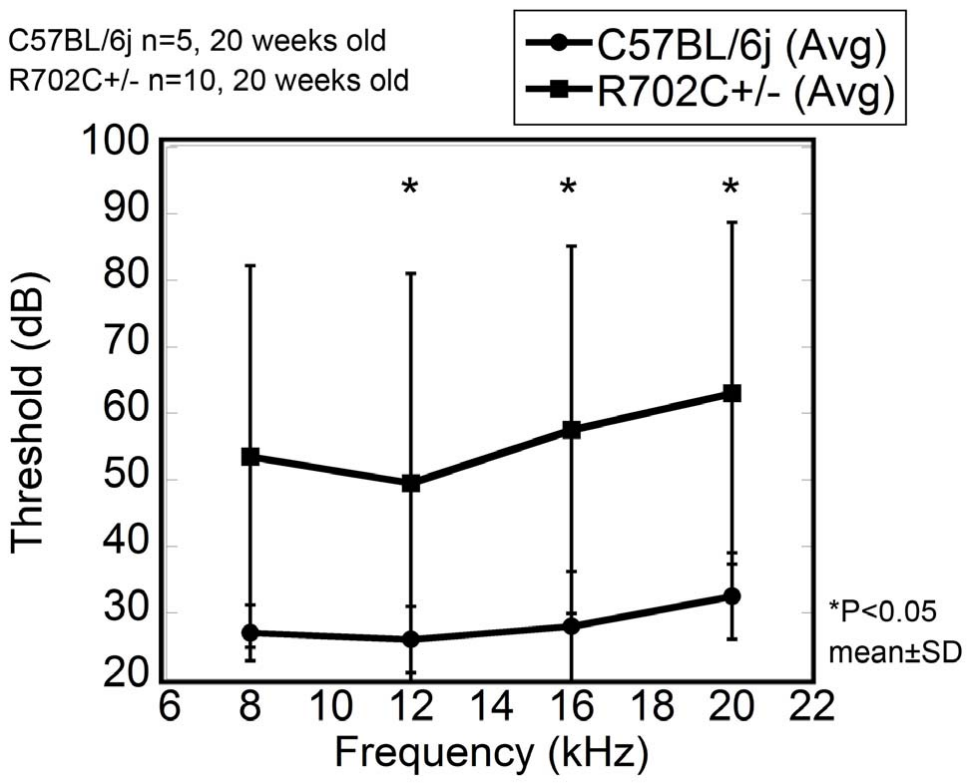
Auditory Brain-stem Response (ABR). ABR measurement in R702C+/− mice. Means and standard deviations of ABR thresholds (in dB SPL) in R702C+/− mice and WT mice. ABRs were measured in 10 R702C+/− mice and five WT mice aged approximately 20 weeks, as described in Materials and Methods.

The mean level of ABR thresholds obtained at 2, 4, 8 and 16 KHz were plotted (Figure 5). WT mice showed normal thresholds for evoke stimuli, whereas the thresholds of ABR in R702C+/− mice were significantly higher at all frequencies. No morphological changes in the inner ears of R702C+/− mice were observed by light or electron microscopy (data not shown).

### R702C+/− mice do not exhibit cataracts

The lenses of six of eight R702C+/− mice showed vacuole formation in the lens fibra, a change characteristic of cataracts (Figure S3). However, the degree of vacuolation was not severe, and indeed, four of six age-matched WT mice also showed similar pathological findings (data not shown).

## Discussion

Zhang et al. generated and characterized three mutant *Myh9* mouse lines: D1424N, E1841K and R702C [11]. D1424N and E1841K mice were generated using the conventional knock-in technique that we employed, but their R702C hetero mice were generated by disrupting the *Myh9* exon 2 and replacing it with human NMMHCIIA fused to eGFP (GFP-R702C mice). On the whole, our R702C+/− mice had clinical phenotypes similar to GFP-R702C hetero mice and both transgenic mouse models reproduced clinical characteristics of *MYH9* disorders. However, the differences in knock-in strategies had lead to differences in intensity of phenocopy expression. Our mice were more definitive in phenocopy than GFP-R702C hetero mice (Table S2).

Especially, R702C+/− mice displayed lower platelet number than D1424N, E1841K and GFP-R702C hetero mice (Table S2). Especially, platelet count was similar to that of megakaryocyte-specific *Myh9* knockout mice with no NMMHCIIA expression in megakaryocytes (*MYH9*Δ mice) [15]. Although platelet diameters were measured under different conditions, platelets of R702C+/− mice were larger than in GFP-R702C hetero mice (Table S2). In humans, it has been reported that R702 mutations provide significantly larger platelets than other mutations located in the tail domain [8]. R702C+/− mice also displayed larger platelet diameters than D1424N and E1841K hetero mice (Table S2), suggesting that our R702C+/− mice mirror the platelet phenotype found in human *MYH9* disorders.

The number of megakaryocytes in R702C+/− mice was increased when compared to that of WT mice (Figure S1). This might be due to reactive thrombocytopoiesis arising from decreased platelet number. The megakaryocyte morphology of R702C+/− mice was similar to that of WT mice (Figure 3A and B) and there were no significant differences in NMMHCIIA localization between R702C+/− and WT mice. However, in human subjects, immunofluorescence analysis using antibodies specific for mutant NMMHCIIA revealed abnormal NMMHCIIA localization in megakaryocytes derived from peripheral blood CD34+ cells of patients with *MYH9* disorders, and these were coarsely and heterogeneously distributed in *MYH9* disorders [16]. This observation indicates that the normal distribution of NMMHCIIA displayed by antibodies against wild-type NMMHCIIA does not necessarily imply that abnormal NMMHCIIA harboring R702C mutation has a normal distribution in megakaryocytes.

It has been proposed that myosin activation through the Rho-ROCK-myosin light chain (MLC)-myosinII pathway could act as a negative regulator of proplatelet formation [17], [18]. NMMHCIIA produced by mice carrying the R702C mutation has weaker affinity for binding ATP and actin than NMMHCIIA in wild-type mice [19]. NMMHCIIA from R702C mice shows a dominant negative effect; proplatelet formation was impaired more severely in R702C+/− mice than in *Myh9* knockout hetero mice showing characteristics of haploinsufficiency [10], [15]. Consequently, *MYH9* disorders may cause premature platelets to be released into the bone marrow due to end stage defects in mature megakaryocytes.

R702C+/− mice showed no obvious changes in bleeding time or clot retraction when compared to WT mice. In human *MYH9* disorders, mutant NMMHCIIA is not transported from megakaryocytes into platelets and it is not present within platelets [16]. Patients with *MYH9* disorders usually exhibit normal primary hemostasis, perhaps because their platelets retain normal function due to residual normal NMMHCIIA. This may be also the case in R702C+/− mice.

In the kidney, R702C+/− mice displayed significant age-dependent albuminuria and glomerulosclerosis. One characteristic was that R702C+/− mice had more global glomerulosclerosis than segmental sclerosis. However, human patients carrying the R702 mutations were reported to display focal segmental glomerulosclerosis (FSGS) [7]. Thus, there were some differences in renal pathogenesis between R702C+/− mice and human patients. In addition, R702C+/− mice displayed little hematuria (Table S1). It was unlikely in view of severe glomerulosclerosis, as human FSGS is known to present with microscopic hematuria. Concerning that our urine test papers were known to work on mouse urine [20], there appears to be some species differences in glomerulopathy caused by R702C mutation. In this context, GFP-R702C hetero mice was reported to display FSGS [11] and this pathological difference may be also caused by a variation in knock-in construct or expression of human/mouse mutated genes.

Johnstone et al. hypothesized that *MYH9* dysfunction in podocytes causes kidney disease in patients with *MYH9* disorders. However, podocyte-specific deletion of *MYH9* in C57BL/6 mice did not evoke kidney dysfunction, although these mice were predisposed to focal and segmental glomerulosclerosis with glomerular changes ranging from mild segmental sclerosis to severe global sclerosis after additional treatment with doxorubicin, used to stress podocytes [21]. These results indicate that overt glomerulosclerosis cannot be caused solely by deletion of *Myh9* in podocytes in mice. NMMHCIIA encoded by *MYH9* is expressed in podocytes and other structures in kidney, such as renal tubular cells, endothelial cells and endocapillary cells [7], [21], [22]. Thus, absence of NMMHCIIA in structures other than podocytes could affect the development of glomerulosclerosis. It is also known that FSGS involves podocytes, as all identified causative genes in hereditary FSGS are located in podocytes [23]. This factor suggests that the R702C mutation that acts on podocytes in a dominant negatively fashion plays an important role in the development of glomerulosclerosis.

In patients with the R702C mutation, granulocyte inclusion bodies are invisible or inconspicuous by MGG staining, although immunofluorescence analysis shows that NMMHCIIA proteins aggregate and accumulate within the granulocyte cytoplasm [8], [16]. R702C+/− mice showed similar characteristics.

Patients with the R702C mutation develop sensory deafness by the age of 30 [7]. ABR experiments showed that around 40% of R702C+/− mice displayed severe deafness, although no abnormalities in cochlear cells were observed by light or electron microscopy (data not shown). Further investigations into how *MYH9* dysfunction results in deafness are required.

R702C+/− mice and WT mice showed similar incidences of cataracts; thus, R702C+/− mice cannot be characterized by the development of cataracts. It is uncertain whether patients with the R702C mutation are predisposed to developing cataracts.

It is unclear why R702C homozygous mice die at the embryonic stage. GFP-R702C mice died at E11.5 because of defects in placental development [11], but R702C+/− mice at E18.5 show no abnormalities in the placenta or umbilical cord (data not shown). Such difference is another example of different construct design, but it requires further exploration to understand how R702C influences embryonic development.

In summary, clinical phenotypes were expressed more grossly in mice by changing the knock-in strategy to exactly represent the phenotype of R702C mutation. Detailed analysis of R702C+/− mice suggested that this mutation causes more severe macrothrombocytopenia and nephritis than tail domain mutations in mice, as well as in humans. Thus, R702C+/− mice will aid our understanding of this complicated human disease.

## Supporting Information

Figure S1 The number of megakaryocyte. Sections of bone marrow samples collected from age matched WT and R702C+/− mice were stained with hematoxylin-eosin. The number of megakaryocytes was counted in 5 fields of view and represent mean±SD (n = 3). Megakaryocyte numbers were increased in R702C+/− mice.
(PPT)

Figure S2 Megakaryocytes with proplatelets. The percentage of megakaryocytes producing proplatelets was determined by the number of megakariocytes with proplatelets. Fetal liver-derived megakaryocytes were examined in suspension cultures. The number of megakaryocytes was counted under a microscope.
(PPT)

Figure S3 Vacuolation of lens fibra (cataract). The pathology of lens of R702C+/− mice and WT mice at 20 weeks stained with Hematoxilin-Eosin. Several samples of R702C+/−mice show vacuolation of lens fibra just below the epithelium lentis.
(PPT)

Table S1 Genotypes of offspring from heterozygous mating.
(PPT)

Table S2 Differences between R702C+/− mice and GFP-R702C hetero mice.
(PPT)
