# Supplementary figures and images for: Establishment of Mouse Model of MYH9 Disorders: Heterozygous R702C Mutation Provokes Macrothrombocytopenia with Leukocyte Inclusion Bodies, Renal Glomerulosclerosis and Hearing Disability
Source: PLoS One. 2013 Aug 20;8(8):e71187. doi: 10.1371/journal.pone.0071187 (PMC3748045; doi:10.1371/journal.pone.0071187)

## Slide 1
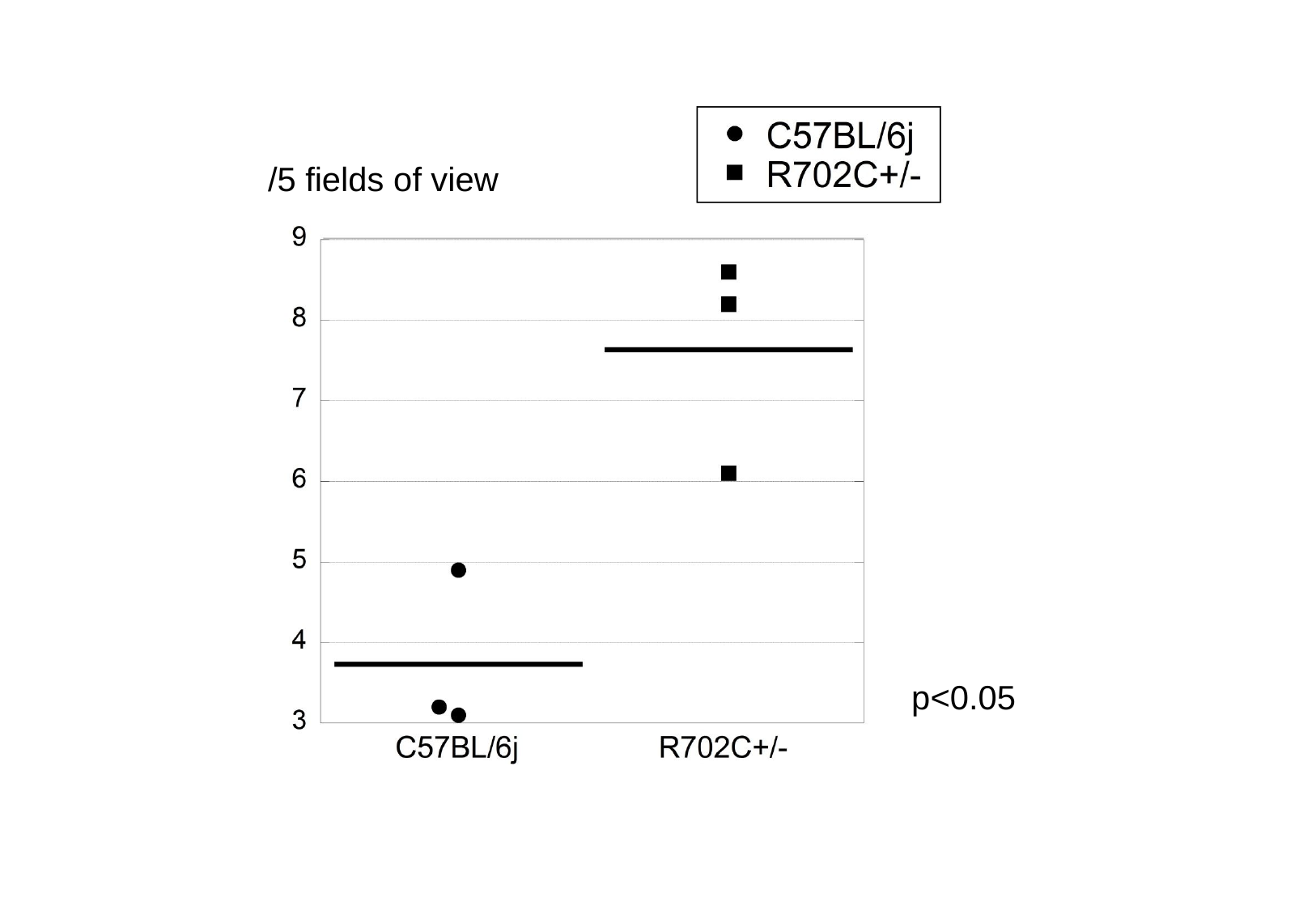

/5 fields of view
p<0.05

Supplement: Figure S1 — The number of megakaryocyte. Sections of bone marrow samples collected from age matched WT and R702C+/− mice were stained with hematoxylin-eosin. The number of megakaryocytes was counted in 5 fields of view and represent mean±SD (n = 3). Megakaryocyte numbers were increased in R702C+/− mice. (PPT) [file pone.0071187.s001.ppt]

## Slide 1
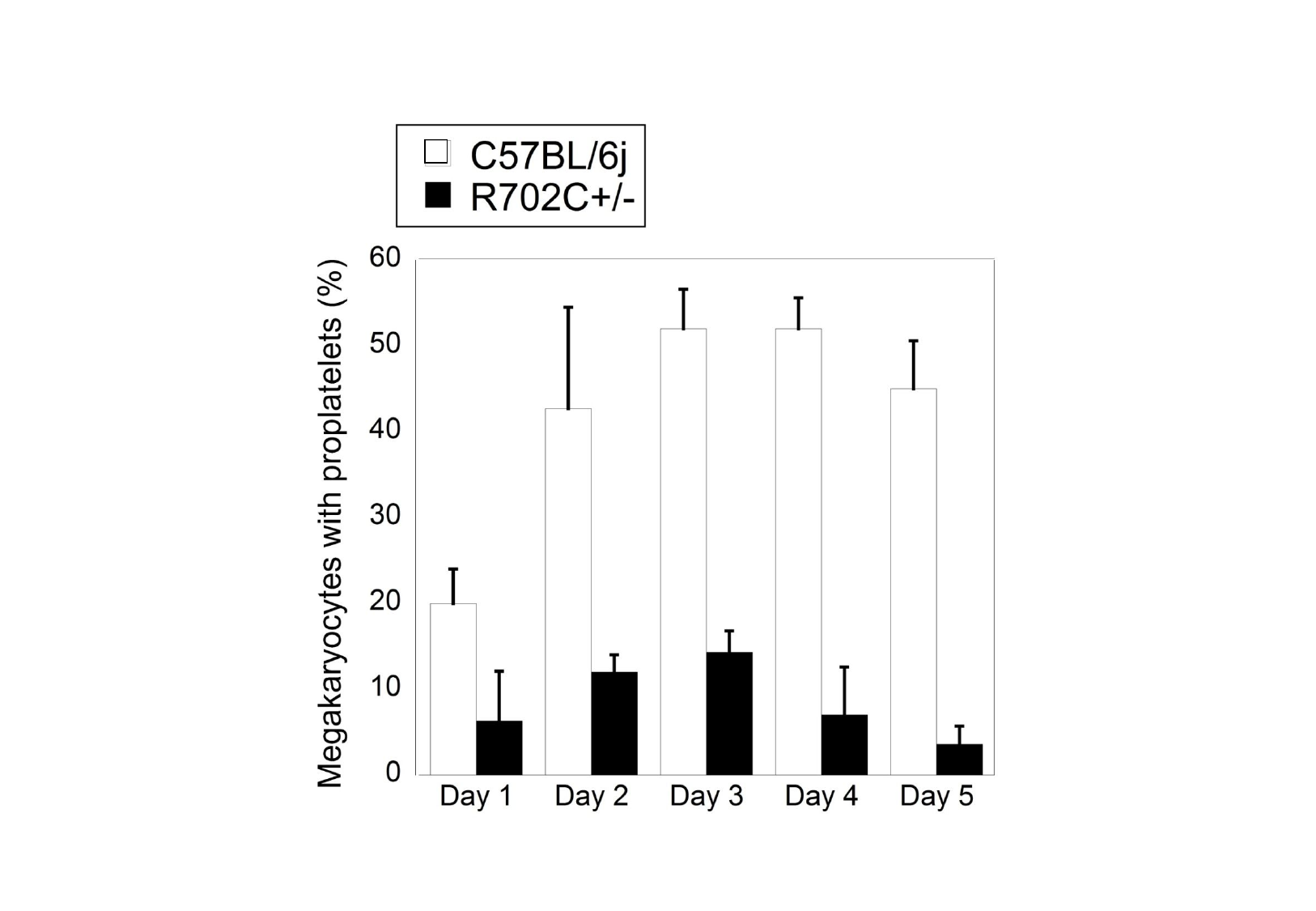

Supplement: Figure S2 — Megakaryocytes with proplatelets. The percentage of megakaryocytes producing proplatelets was determined by the number of megakariocytes with proplatelets. Fetal liver-derived megakaryocytes were examined in suspension cultures. The number of megakaryocytes was counted under a microscope. (PPT) [file pone.0071187.s002.ppt]

## Slide 1
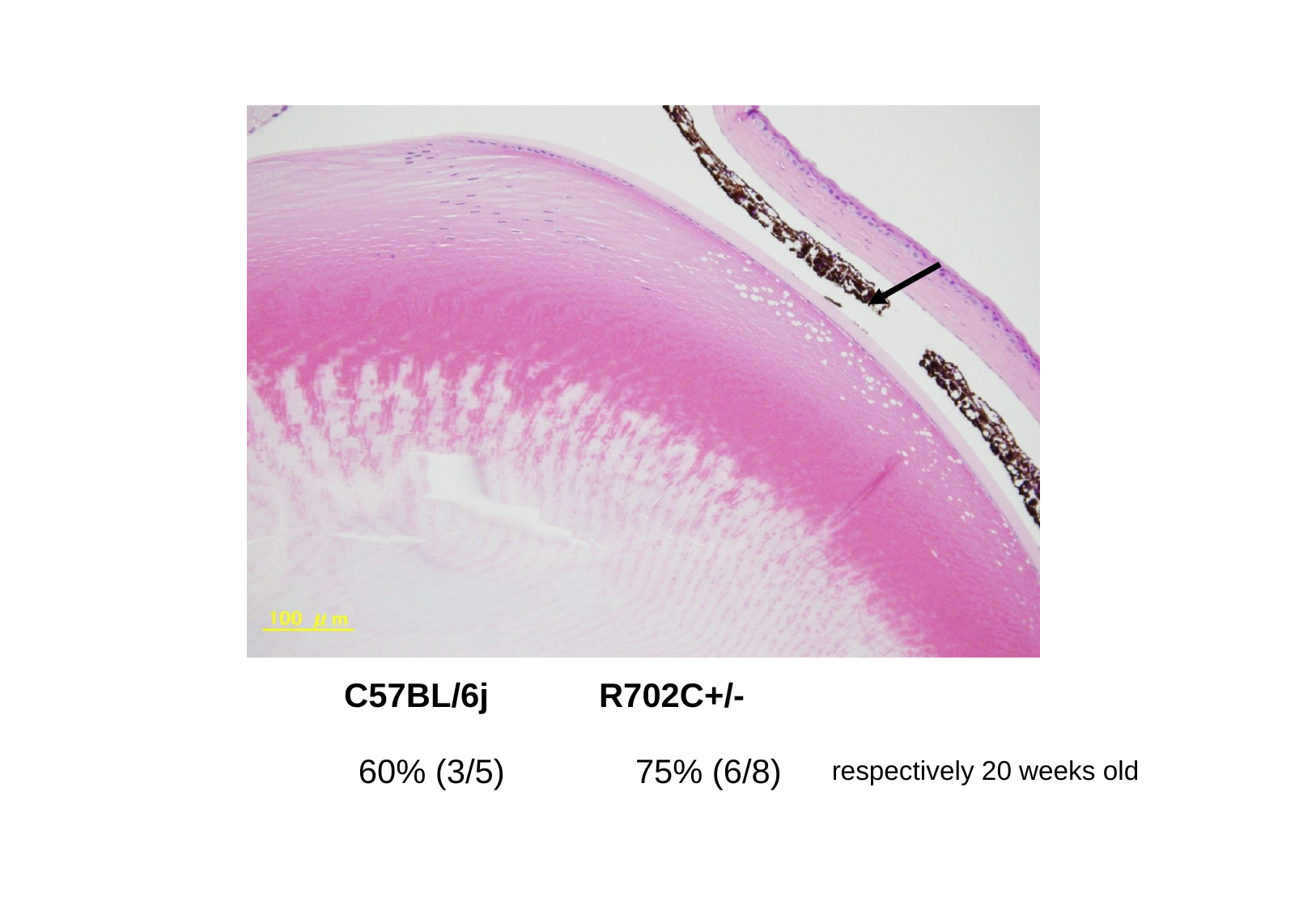

| C57BL/6j | R702C+/- |
| --- | --- |
| 60% (3/5) | 75% (6/8) |
respectively 20 weeks old

Supplement: Figure S3 — Vacuolation of lens fibra (cataract). The pathology of lens of R702C+/− mice and WT mice at 20 weeks stained with Hematoxilin-Eosin. Several samples of R702C+/−mice show vacuolation of lens fibra just below the epithelium lentis. (PPT) [file pone.0071187.s003.ppt]
