# Supplementary material for: Establishment of Mouse Model of MYH9 Disorders: Heterozygous R702C Mutation Provokes Macrothrombocytopenia with Leukocyte Inclusion Bodies, Renal Glomerulosclerosis and Hearing Disability
Source: PLoS One. 2013 Aug 20;8(8):e71187. doi: 10.1371/journal.pone.0071187 (PMC3748045; doi:10.1371/journal.pone.0071187)
Supplement: Table S1 — Genotypes of offspring from heterozygous mating. (PPT) [file pone.0071187.s004.ppt]

## Slide 1
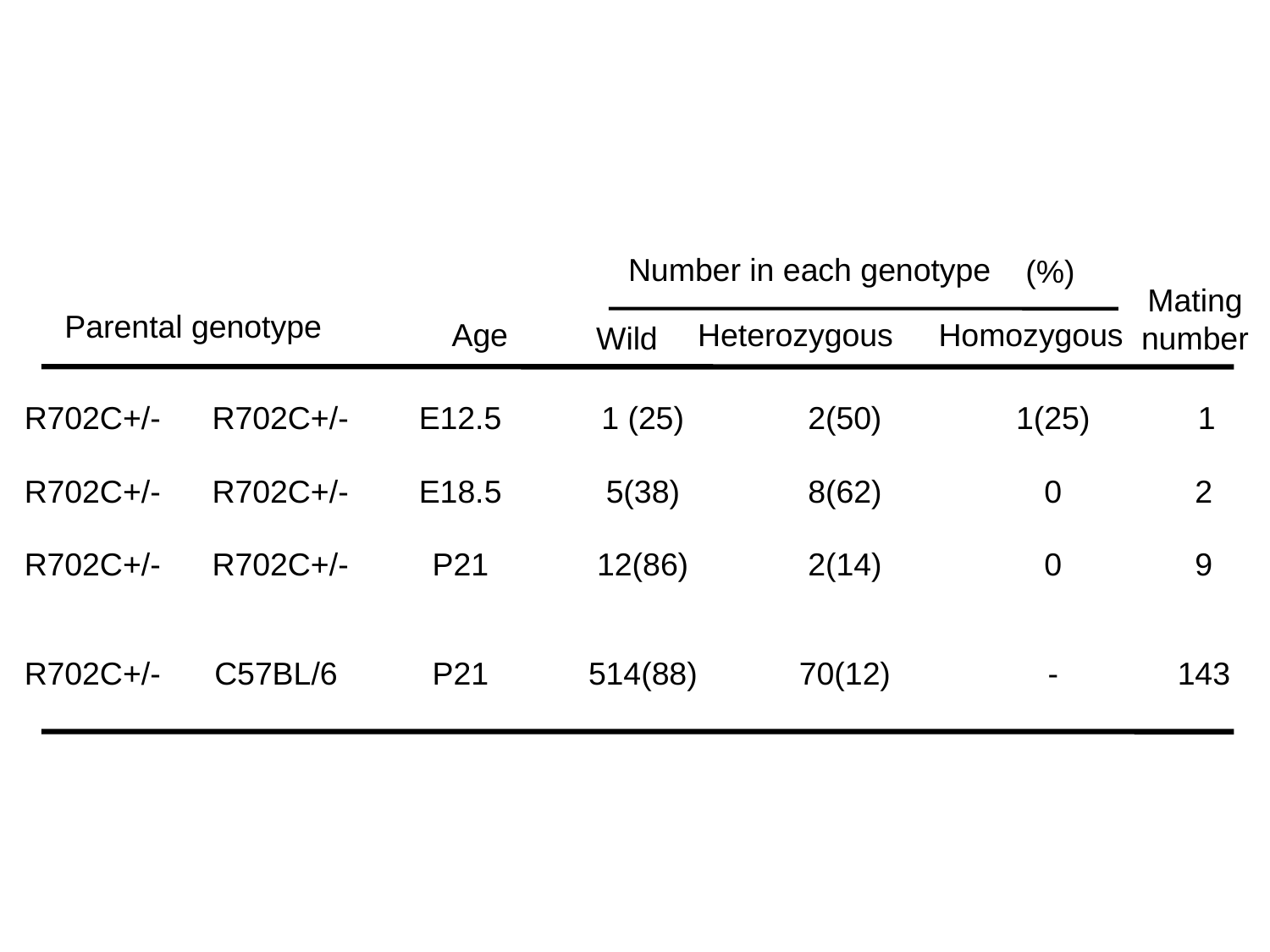

Number in each genotype
(%)
Mating number
Parental genotype
Age
Heterozygous
Homozygous
Wild
R702C+/-
R702C+/-
E12.5
1 (25)
2(50)
1(25)
1
R702C+/-
R702C+/-
E18.5
5(38)
8(62)
0
2
R702C+/-
R702C+/-
P21
12(86)
2(14)
0
9
R702C+/-
C57BL/6
P21
514(88)
70(12)
-
143
