# Supplementary material for: Establishment of Mouse Model of MYH9 Disorders: Heterozygous R702C Mutation Provokes Macrothrombocytopenia with Leukocyte Inclusion Bodies, Renal Glomerulosclerosis and Hearing Disability
Source: PLoS One. 2013 Aug 20;8(8):e71187. doi: 10.1371/journal.pone.0071187 (PMC3748045; doi:10.1371/journal.pone.0071187)
Supplement: Table S2 — Differences between R702C+/− mice and GFP-R702C hetero mice. (PPT) [file pone.0071187.s005.ppt]

## Slide 1
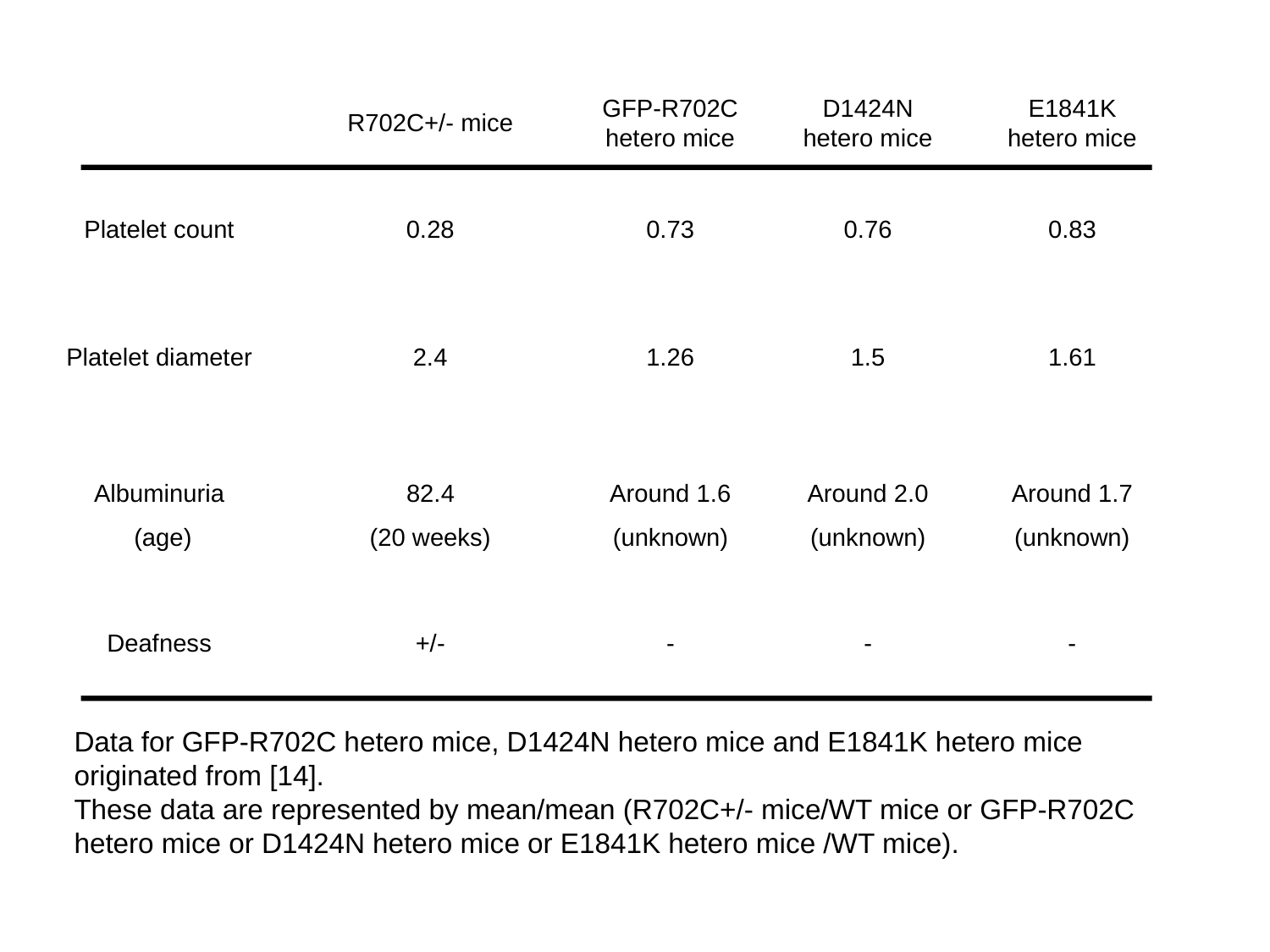

GFP-R702C hetero mice
D1424N hetero mice
E1841K hetero mice
R702C+/- mice
Platelet count
0.28
0.73
0.76
0.83
Platelet diameter
2.4
1.26
1.5
1.61
Albuminuria
82.4
Around 1.6
Around 2.0
Around 1.7
(age)
(20 weeks)
(unknown)
(unknown)
(unknown)
Deafness
+/-
-
-
-
Data for GFP-R702C hetero mice, D1424N hetero mice and E1841K hetero mice originated from [14].
These data are represented by mean/mean (R702C+/- mice/WT mice or GFP-R702C hetero mice or D1424N hetero mice or E1841K hetero mice /WT mice).
